# Social participation in the city: exploring the moderating effect of walkability on the associations between active mobility, neighborhood perceptions, and social activities in urban adults

**DOI:** 10.1186/s12889-023-17366-0

**Published:** 2023-12-07

**Authors:** Lukas Bollenbach, Christina Niermann, Julian Schmitz, Martina Kanning

**Affiliations:** 1 Department of Social and Health Sciences in Sport Science, University of Konstanz, Konstanz, Germany; 2 Institute of Interdisciplinary Exercise Science and Sports Medicine, Medical School Hamburg, Hamburg, Germany; 3 Research Institute for Regional and Urban Development gGmbH, Dortmund, Germany

**Keywords:** Urban environment, Social Participation, Active mobility, Subjective neighborhood perceptions, Walkability, Physical activity, Environmental perceptions, Social activities, City, Cross-sectional

## Abstract

**Background:**

Living in urban environments is associated with several health risks (e.g., noise, and air pollution). However, there are also beneficial aspects such as various opportunities for social activities, which might increase levels of social participation and (physically) active mobility that in turn have positive effects on health and well-being. However, how aspects of the environment, active mobility, and social participation are associated is not well established. This study investigates the moderating effect of low vs. high walkability neighborhoods on the associations between active mobility, and social participation and integrates individuals’ subjective perception of the neighborhood environment they are living in.

**Methods:**

Cross-sectional data from 219 adults (48% female, mean age = 46 ± 3.8 years) from 12 urban neighborhoods (six low, six high walkability) were analyzed: First, social participation, active mobility, and subjective neighborhood perceptions were compared between people living in a low vs. high walkability neighborhood via t-tests. Second, multigroup path analyses were computed to explore potential differences in the associations between these variables in low vs. high walkability neighborhoods.

**Results:**

Social participation, active mobility, and subjective neighborhood perceptions didn’t differ in low vs. high walkability neighborhoods (p: 0.37 − 0.71). Active mobility and subjective neighborhood perceptions were significantly stronger related to social participation in low vs. high walkability neighborhoods (active mobility in low: *ß* = 0.35, p < .01 vs. high: *ß* = 0.09, p = .36; subjective neighborhood perceptions in low: *ß* = 0.27, p < .01 vs. high: *ß* = 0.15, p = .18).

**Conclusions:**

Despite living in neighborhoods with objectively different walkability, participants rated social participation and active mobility equally and perceived their neighborhoods similarly. However, zooming into the interrelations of these variables reveals that social participation of residents from low walkability neighborhoods depends stronger on active mobility and perceiving the environment positively. Positive perceptions of the environment and active mobility might buffer the objectively worse walkability. Future research should focus on underlying mechanisms and determinants of subjective neighborhood perceptions and active mobility, especially in low walkability neighborhoods.

**Supplementary Information:**

The online version contains supplementary material available at 10.1186/s12889-023-17366-0.

## Background

Living in an urban environment has been shown to be negatively associated with health and well-being, for example via noise and air pollution, but also via social-isolation [1–3]. Besides social isolation, globally low rates of physical activity are a public health concern that also apply to urban populations [4]. Consequently, and in light of continuing urbanization, the realization of healthy urban neighborhoods that promote social participation, physical activities (including walking and biking for transport and recreation, = active mobility), and ultimately the health of all their residents are important [1, 4–7]. However, research in the context mostly concerns characteristics of the built or natural environment for physical activities: For example, a systematic review conflated what the environment needs to look like or how it needs to be modified to increase levels of physical activities and reduce obesity [8]. Aspects of the social environment like social participation, on the other hand, are often neglected or only regarded as concomitant. Despite the emphasized importance of reducing negative social environmental aspects such as social isolation, they still “(…) have generally been underrecognized and underappreciated relative to the evidence supporting their public health importance” ( [9], p. 55).

Research supports the importance of individuals’ social environment for their health and well-being: For example, even meeting someone on the street can improve momentary affective states [10, 11]. Also, support from friends or family can act as a protective health resource and aid in promoting various health outcomes (e.g., happiness, life satisfaction, and well-being) [12, 13]. In addition, participating in social activities like meeting with close ones is positively associated with physical and mental health [14, 15]. Furthermore, interaction with other people can improve momentary health such as less tiredness and sadness and more happiness [16]. It becomes apparent that many different aspects concerning individuals’ social environment are being researched, which makes it difficult to synthesize results. What different concepts (e.g., social interaction, social capital) have in common is that they indicate or describe a certain amount- or lack of social participation, which is defined “(…) as the involvement of the person in activities that provide interactions with others in the community (…)” ( [17], p. 1718), [18]. For this reason, social participation is used as the overarching concept in this work. However, little is known about the influence of the context in which social participation takes place. For example, how do residents perceive the presence of other individuals, traffic, or greenness, and does the interaction take place in a narrow urban alley, a marketplace, or are there a lot of high-rises? Accordingly, the most promising avenue to understand how to promote social participation and health in urban neighborhoods is to incorporate the individual, the social-, and the built environment, and thus all facets of urban life [19–21].

In this regard, an important individual correlate is active mobility: Higher levels of active mobility have been associated with increased social interaction [22] - on a side note, it is further associated with physical activity, and can help to reduce traffic and concomitant air pollution [22–24]. Still, more knowledge about how active mobility and neighborhood environments function together, especially in neighborhoods with different characteristics, to support or facilitate increased levels of social participation, and ultimately health, is needed. Social participation is further conceptualized as a result of the two-way interplay between individuals and environmental factors [17, 25]. Therefore, environmental factors need to be measured and accounted for. Both the design and the perceptions of neighborhoods have an impact on residents’ satisfaction and well-being [26]. Residents’ perception of- and satisfaction with the neighborhood environment play an important role regarding social participation: For example, increased satisfaction of residents with their neighborhood regarding connectivity and availability of amenities is associated with increased social participation and engaging in activities with people of the same residential area is correlated with both satisfaction and attachment with that residential area [27]. It follows that the perceptions of- and the built environment itself are key correlates of social participation, as they can not only in- or decrease the possibility of engaging in them but also determine to a great extent the equal access to social- and recreational activities that are essential for healthy neighborhoods [27–29]. In this context, cities that offer accessible mixed-usage areas, have a high density and good connectivity, and are safe, are claimed to lead to more social interactions, active mobility, and increased livability [30, 31]. In addition, easily accessible amenities, greenspaces, and parks have been linked to increased levels of physical activity and active mobility [32].

However, neighborhoods often vary regarding specific features and characteristics (e.g., topography, layout, amenities, etc.). In this context, the concept ‘walkability’ is widely used to describe the accessibility and friendliness of environments in facilitating active mobility (e.g., [33, 34]). High walkability indicates a high friendliness for active mobility, whereas low walkability indicates reduced/low friendliness. For example, walkability can include measures of amenities, parks, and other places, it can incorporate crosswalks, pedestrian streets, etc., but it can also be calculated via sub-components like residential density, connectivity, and land use mix (for an overview see [35]). These measures can also increase the probability of individuals engaging in social participation. For example, walkability has been used to describe associations between environments and social participation [36, 37]. While the value of using walkability in describing and comparing urban areas is well documented (e.g., [35]), it has to be mentioned that also a few discrepancies regarding the concordance of walkability assessments in North America/Australia compared with European ones were found [38]. However, walkability can be used to determine how a specific urban area supports individuals in being physically active in everyday life (e.g., [39]). With that, it can also help to increase levels of physical activity and active mobility and through this help to promote public health [40]. There are different approaches to collecting this information, for example objectively via data from geographic information systems (GIS) or subjectively via data from surveys of individuals via self-report, with research recommending to include both when studying residential areas [26]. It’s important to highlight that the objective walkability provides one measure that applies to all individuals living in a specific area (i.e., the same measure for everyone). This enables comparisons of built environments. Contrary to this, self-reports provide an individual measure for every single person and can therefore vary, even if persons live in the same area.

In sum, the concept walkability implies an objective categorization into low and high walkable residential areas and allows comparison between them with regard to other individuals’ characteristics like subjective neighborhood perceptions, active mobility, and social participation. Still, most research doesn’t investigate these aspects together and focuses on more isolated aspects of either physical activities, active mobility (e.g., [41]), the (built) environment (e.g., [42]), or social participation (e.g., [17]). Hence, this study integrates social participation, active mobility, and neighborhood characteristics to investigate and describe the interdependencies between them. The aims of the study are (1) to examine differences between levels of active mobility, subjective neighborhood perceptions, and social participation depending on living in a high or low walkability neighborhood and (2) to explore whether the associations between subjective neighborhood perceptions, active mobility, and social participation differ depending on low vs. high walkability.

## Method

### Study design

A cross-sectional online questionnaire was conducted and implemented via the German online platform ‘SoSci Survey’ [43]. The data were collected between July and December 2020 in the city of Stuttgart, Germany. For clarification, data collection took time during the COVID-19 pandemic, but no serious restrictions (e.g. curfews) were in place in the data collection timeframe. The first page of the questionnaire contained information about the study, its goals, data privacy protection, and participants’ rights in this context. To participate in the study, participants had to give informed consent that they were willing to participate and had read and understood the study information. However, this study includes only a part of all collected data (see ‘Measures’). The survey was in German.

### Recruitment of the study participants

**Fig. 1:**
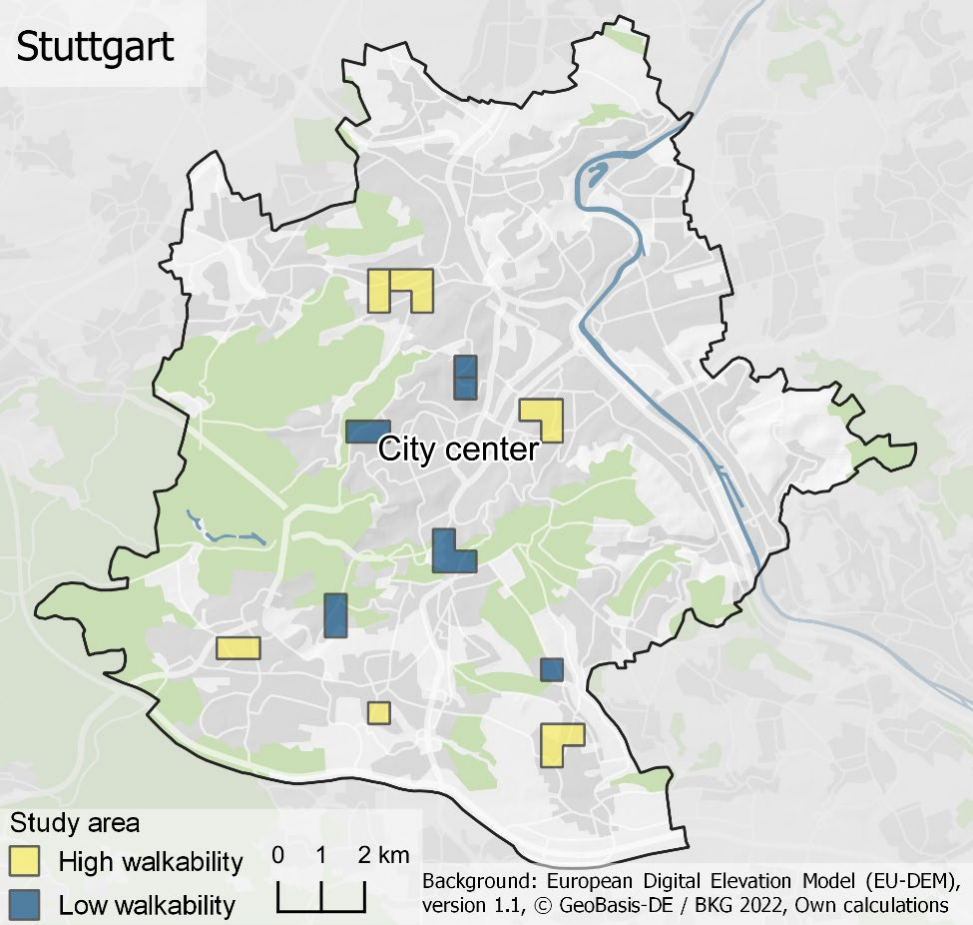
Study area with the 12 pre-selected residential neighborhoods (six low and six high walkability neighborhoods)

The individuals who participated in this study were recruited via the distribution of 3000 letters in 12 pre-selected residential areas in the city of Stuttgart, Germany. The letters contained information about the study background, a QR code to directly participate in the online questionnaire, and information about the option to participate via a paper-pencil questionnaire. Inclusion criteria were to live in the study area, to be at least 18 years old, and to understand German. The study sample is described in Table 1. The residential areas were pre-selected based on the objective walkability in the respective residential area, resulting in six residential areas with low walkability, and six residential areas with high walkability (see Fig. 1). The walkability scores for the classification of the pre-selected areas into low- and high walkability were derived via the first version of the ILS-Walkability-Index [35].

### Measures

The following measures were derived and used in the data analyses to answer the research questions.

### Social participation

To measure social participation, the scale used by Levasseur et al. [17] was adopted, which operationalizes social participation as participants’ frequency of monthly engagement in 10 different social activities. The response options to the question “How often are you involved in the following activities?” were rated on a 5-point Likert scale with the following indications: 1 (“never”), 2 (“less than once a month”), 3 (“at least once a month”), 4 (“at least once a week”), and 5 (“almost every day”). After data collection, the response options were converted into frequencies per month per activity (“almost every day” = 20; “at least once a week” = 6; “at least once a month” = 2; “less than once a month” = 1; and “never” = 0, respectively). In a final step, the frequencies from all 10 activities were summed, which resulted in the final social participation score that constitutes the number of social activities per individual per month with a theoretical range of 0-200 (note, it is hardly possible to be involved in every social activity on every day) [17]. One example for the question and a response option is as follows (see ‘Additional file 3’ for further information): ‘How often are you involved in the following activities?’

Visit family members/friends.

### Active mobility

To assess individuals’ level of active mobility, the validated ‘Physical Activity, Exercise, and Sport Questionnaire’ was used [44]. The questionnaire assesses various types of physical activities (such as everyday life activities, e.g., walking/cycling to work or leisure, household activities), exercises (for the purpose of physical activity itself, e.g., running, hiking), and sports (a more specific sport, often with a competitive character, e.g., soccer, track and field athletics). However, as for this study only walking and bicycling to work, for leisure, and for recreational purposes (active mobility) was of interest, only these measures were utilized. This resulted in a total of five items (1, walking to work; 2, walking to the grocery store; 3, bicycling to work; 4, bicycling for other transportation purposes; and 5, walking for recreation/strolling) that were summed up to the measure of active mobility. The items were assessed in the following manner [44]. After the introduction question “On how many days, and for how long have you conducted the following activities in the last four weeks?”, participants answered in cloze-type-questions, for example (see ‘Additional file 1’ for further information):

Walking to work (also partial sections): On _ days during the 4 weeks and approximately _ minutes per day.

With the information from the first (number of days of the respective activity) and the second (performed minutes per respective activity) response, the active mobility per month per participant (unit: minutes of active mobility per month per participant) was calculated and used in the analyses. This was done by summing up the products from each multiplication of days and minutes for 1, 2, 3, 4, and 5, respectively.

### Neighborhood perceptions

Participants’ subjective neighborhood perceptions, i.e., their subjectively perceived satisfaction with the neighborhood environment, were measured via selected questions from the validated ‘Neighborhood Environment Walkability Scale - Germany’ (NEWS-G [45, 46]). To be precise, 10 questions from the subcategory ‘I’ (‘satisfaction with the neighborhood environment’) were assessed (see ‘Additional file 2’). The participants answered the questions regarding their satisfaction with different environmental features on a 5-point Likert scale, with answers ranging from 1 (“very unsatisfied”) to 5 (“very satisfied”). The final scale for analyses resulted from the mean of the answers. The scale had acceptable reliability with a Cronbach’s Alpha of 0.74. One example for a question and response is as follows (see ‘Additional file 2’ for further information): ‘How satisfied are you with…’

… the possibility to walk in your neighborhood environment?

Note that ‘subjectively perceived satisfaction with the neighborhood environment’ is abbreviated to ‘neighborhood perceptions’ in the rest of the manuscript to increase readability.

### Walkability

First, the walkability measure that was used for the initial pre-selection of the 12 residential areas for participant recruitment was rechecked and updated with an adapted and improved version of the walkability measure that wasn’t available at the time of the initial data collection. We used the Walkability-Index from the Research Institute for Regional and Urban Development (= ‘Institut für Landes- und Stadtentwicklungsforschung’, ILS; ‘ILS-Walkability-Index’) to measure the objective walkability. The index was refined in the project ‘AMbit - Active Mobility’ [47] and is based on the basic concept of the original Walkability-Index, which was developed by Dobešová and Křivka [39]. We used new technical possibilities such as precise routing and open data [35]. Generation of the measure was done as follows: This objective walkability for the city of Stuttgart was determined using QGIS (a free and Open Source Geographic Information System software) to calculate the ILS-Walkability Index [35]. We calculated the walkability city-wide on a 500m by 500m grid and checked in which grid the participants live. For each cell of the grid, a score was calculated. The ILS-Walkability-Index consists of four dimensions: The permeability of the pedestrian network (data source: OpenStreetMap, European Digital Elevation Model), the proportion of green spaces (data source: OpenStreetMap), the population density (data source: German Zensus, 2011), and the availability of amenities (data source: OpenStreetMap) within walking distance. The permeability of the pedestrian network shows the area that a person can reach when walking 500m in any direction along the pedestrian network starting from the center of each cell. The result is a polygon – the so-called pedestrian shed. It is put in relation to the theoretical maximum size of the pedestrian shed – a circle with a radius of 500m. The higher the proportion is, the more permeable the pedestrian network is. An elevation model serves as a correction factor: The more meters of altitude, the smaller the pedestrian shed is. The proportion of green space is the proportion of the pedestrian shed that is covered with green space. Population density is derived from the number of residents living within the pedestrian shed. The accessibility of amenities is based on calculations of the distance along the walking network to different amenities such as supermarkets, schools, or restaurants. The closer and more numerous the amenities are, the higher the rating is. All four dimensions (permeability of the pedestrian network, green space, population density, amenities) are scaled from 0 to 10 and added together to the ILS-Walkability Score. Because population density correlates with the amenity-score (where many people live, there are a greater number of stores), a weight of 0.5 was applied to population density, while a weight of 1 was applied to the other dimensions. The sum is stretched to a scale from 0 to 50, where 50 represents the maximum walkability. Walkability was then categorized using tertiles. The first tertile includes grids with a range from 33 to 50 and corresponds to a high walkability. The second a range from 23 to 33 and corresponds to average walkability. Lower values correspond to a low walkability. Therefore, a value **≤** 23 indicates low walkability, and a value of **≥** 33 indicates high walkability. For the calculation, we used data from OpenStreetMap [48], the German Zensus 2011 [49], and the European Digital Elevation Model [50] to calculate the altitude. To calculate the distances, we used the OpenRouteService [51] from Heidelberg Institute for Geoinformation Technology [52]. For more details see [35].

### Covariates

The demographics sex, age, height, weight, and socioeconomic status (SES) (Table 1.) were measured via self-report in the questionnaire. The SES represents a multidimensional index score that comprises three continuously measured components ‘Education and Occupational Qualifications’ (highest one achieved, e.g., Higher School Certificate), ‘Occupational Status’ (e.g., civil servant; comparative classification), and ‘Net Income’ that go into the index equivalently [53]. The SES had a possible range of 3–21 and was divided into 5 quintiles (low, 1. quintile, threshold = 6.6 (Q1); medium, 2.–4. quintile, threshold = 10.2 (Q2), 13.8 (Q3), 17.4 (Q4); high, (5. quintile, threshold = 21). Based on the self-reported height and weight, the BMI was calculated for each individual.

### Analyses

**Fig. 2:**
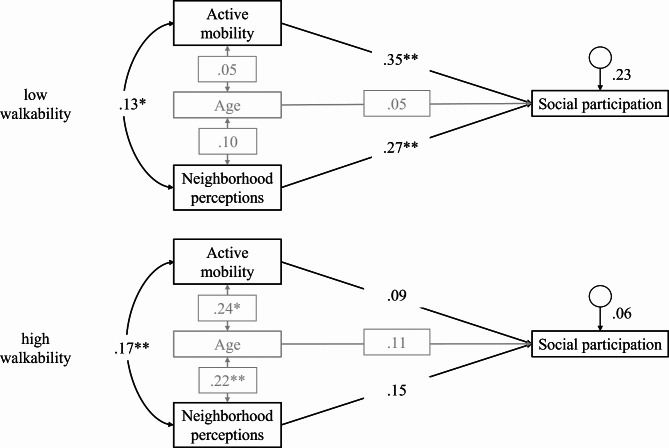
Model 2, (regression weights freely estimated, correlation coefficients constraint equally) for low vs. high walkability. *Note*: Double arrows indicate a correlation, single arrows a regression. Age was integrated as a covariate (grey). * indicates p < .05, **p < .01, and ***p < .001

Data were analyzed using SPSS 27.0 (IBM Corp., NY, USA). Missing values were excluded pair-wise. Due to the violation of the normal distribution of the data, we used bootstrapping to obtain estimates of the standard errors and compute confidence intervals and significance tests. For calculating bias-corrected 95% confidence intervals, 1000 bootstrapping iterations were requested. Multigroup path analyses for three different models with walkability (low vs. high) as a moderator were performed to investigate the associations between subjective neighborhood perceptions (independent variable), active mobility (independent variable), and social participation (dependent variable), and we investigated the necessity to control for age, sex, and SES via correlations, t-tests, including covariate age in the analysis with IBM AMOS 27.0 (IBM Corp., NY, USA). Multigroup path analyses allow to investigate variations in the relations of variables across different groups: Here, a group of people living in high walkability neighborhoods (high walkability group) was compared with a group of people living in low walkability neighborhoods (low walkability group) [54, 55]. Maximum likelihood estimation was used to test the hypothesized sequence of the associations as depicted in Fig. 2. The commonly recommended fit indices χ^2^/df, CFI, and RMSEA were used to assess the goodness of fit. A good fit is indicated by 0 ≤ χ^2^/df ≤ 2, 0.97 ≤ CFI ≤ 1, and RMSEA ≤ 0.05, and an acceptable fit was indicated by 2 < χ^2^/df ≤ 3, 0.95 ≤ CFI < 0.97, and 0.05 < RMSEA ≤ 0.08 [56]. Assuming that regression coefficients differ according to low vs. high walkability of the neighborhood environment, we tested an unconstrained model where all paths (regression weights and correlation coefficients) (model 1) are freely estimated in both samples, a partially constrained model assuming equivalence of correlation coefficients (model 2; Fig. 2), and a fully constrained model assuming equivalence of regression weights and correlation coefficients in the two samples (model 3). We then tested whether the partially constrained model 2 has a significantly better fit (delta chi-square < 0.05) than the fully constrained model 3, and whether the unconstrained model 1 has a significantly better fit than the partially constrained model 2 by performing a chi-square difference test in combination with considering the fit-indices CFI, RMSEA, and AIC [57, 58].

## Results

### Descriptive characteristics

Descriptive characteristics are depicted in Table 1. The overall study population consisted of 219 individuals with a mean age of 46.9 (*SD* = 16.5; 48% female). The average SES of the participants was 14.3 (*SD* = 3.8), resulting in a study population with an upper-medium SES [53].

**Table 1:** Descriptive statistics

*Descriptives*	
Participants (N)	219
Sex	48% female
Age (*M*, SD)	46.89 (± 16.47) years
Height (*M*, SD)	174.84 (± 8.52) cm
Weight (*M*, SD)	73.86 (± 13.50) kg
BMI (*M*, SD)	24.06 (± 3.4)
SES (*M*, SD)	14.29 (± 3.8)

*Note*: *M* = mean; SD = standard deviation; BMI = body mass index; SES = socioeconomic status, range 3 (lowest) − 21 (highest)

Study variables social participation (operationalized via social activities), neighborhood perceptions, and active mobility were correlated with small to moderate effect sizes, correlation coefficients are shown in Table 2.

**Table 2:** Means, standard deviation, range of study variables, and their correlations

*Variables*	*M* (SD)	min/max	Pearson correlation coefficients^a^ *r* (*p*), [95% CI]	
			2	3
1 Social participation	33.85 (± 15.9)	7–88	0.25 (< 0.001) [0.01, 0.34]	0.26 (< 0.001) [0.12, 0.38]
2 Neighborhood perceptions	3.96 (± 0.6)	2.1-5		0.14 (0.05) [0.01, 0.26]
3 Active mobility	1062.16 (± 786.5)	60-4320		

*Note*: *M* = mean; SD = standard deviation; min-max: minimum-maximum; variables: social participation, number of social activities/month/individual; neighborhood perceptions, values 1 (very unsatisfied) − 5 (very satisfied); active mobility, minutes/month/individual; ^a^based on bias-correcting bootstrapping with 1000 samples; CI = confidence intervals

In addition, the following significant correlations between the study variables and age, sex, and SES can be reported. For social participation with age: 0.14, p < .05, and social participation with SES: − 0.19, p < .01. For neighborhood perceptions with age: 0.14, p < .05, and for active mobility with age: 0.15, p < .05.

### Comparison of study population and variables in low vs. high walkable neighborhoods

Of the 219 participants, 100 (47.4%) lived in a low, and 111 (52.6%) lived in a high walkability neighborhood (N = 8 did not provide an answer). The low walkability neighborhoods had a mean value of 21.4 (± 0.6) vs. 38.7 (± 0.1) in high walkability. There were no differences (except for age) between individuals from low and high walkability neighborhoods concerning the demographics (sex, χ^2^ [2] = 1.40, p = .5, low: N = 50 female, high: N = 52 female; age, *t*(*209*) = 3.62, p < .001, low: mean = 51.3 ± 17.4, high: mean = 43.5 ± 14.9; BMI, *t*(*208*) = -1.16, p = .24, low: mean = 23.8 ± 3.3, high: mean = 24.3 ± 3.6; SES, *t*(*208*) = − 0.47, p = .63, low: mean = 14.1 ± 3.6; high: mean = 14.4 ± 4.1). Investigating the relevance of walkability of the different neighborhoods by comparing the means of the study variables between subjects living in low walkability vs. high walkability revealed the following results (Table 3): Multivariate analysis of covariance showed that social participation, active mobility, and neighborhood perceptions did not differ depending on low vs. high walkability (*F*(3, 206) = 0.57, p = .64), but there was a significant effect of age (*F*(3, 206) = 2.89, p = .04).

**Table 3:** Comparison of means (MANCOVA) of social participation, neighborhood perceptions, and active mobility in low vs. high walkability

*Variables*	low walkability	high walkability	*F*(1, 208), *p*,
	*M* (SD)	*M* (SD)	
Social participation	35.44 (± 17.30)	32.30 (± 14.44)	0.80, 0.37
Neighborhood perceptions	3.98 (± 0.57)	3.97 (± 0.57)	0.26, 0.61
Active mobility	1072.73 (± 865.38)	1060.01 (± 750.70)	0.14, 0.71

*Note*: *M* = mean; SD = standard deviation; variables: social participation (number of social activities/month/individual); neighborhood perceptions, values 1 (very unsatisfied) − 5 (very satisfied); active mobility (minutes/month/individual); * p < .05; ** p < .01

### Comparison of relationships between study variables in low vs. high walkable neighborhoods

As the means of the study variables and social-demographic variables didn’t differ between the groups and as the results of the t-tests for the possible confounders age, SES, and sex showed only significant differences for age between the groups, we controlled for age in the analysis. Investigating whether there was a significant difference in the relationship between active mobility and neighborhood perceptions, and how they predicted social participation depending on individuals living in low vs. high walkability, showed the following results (Table 4). While the χ^2^ difference test (Δχ^2^: 5.97 − 0.03 = 5.94, *df*: 3 − 1 = 2; p = .05) by itself didn’t show that the partially constrained model 2 (freely estimated regression weights) had a better model fit than fully constrained model 3 (equivalence of regression weights and correlation coefficients), the fit indices CFI, RMSEA, and AIC did: The CFI for model 3 was 0.92, while the CFI for the model 2 was 1, showing an improvement in CFI of 0.08; the RMSEA for model 3 was 0.069, while RMSEA for model 2 was 0.000, showing an improvement in RMSEA of − 0.069; and the AIC for model 3 was 55.97, while AIC for model 2 was 54,02, showing an improvement of 1.93. This indicates that the regression weights differ depending on low and high walkability. In addition, model 1 (unconstrained model) revealed no additional gain compared to model 2 (partially constrained) (Δχ^2^: 0.02, *df* = 1; p = .16).

**Table 4:** Fit indices of the unconstrained (1), partially constrained (2), and fully constrained model (3)

Models	χ2	df	*p*	χ2/df	CFI	RMSEA	90% CI	AIC
3	5.97	3	0.11	1.99	0.92	0.06	0.00-0.15	55.97
2	0.02	1	0.87	0.02	1.00	0.00	0.00-0.09	54.02
1	0.00	0	-	-	1.00	0.12	0.08-0.16	56.00

*Note*: χ2 = Chi-square; df = degrees of freedom; CFI = Comparative Fit Index; RMSEA = Root Mean Square Error of Approximation; CI = Confidence Intervals; AIC = Akaike Information Criterion. Adjusted for age

Thus, the relationship between the investigated variables was significantly different in low walkability compared to high walkability. In addition, Fig. 2 illustrates model 2 with the best model fit and shows the correlation of neighborhood perceptions and active mobility and the regression paths regarding the prediction of social participation. Age was integrated as a covariate. The results show that while the correlation coefficient between active mobility and neighborhood perceptions with *r* = .13 (95% CI [0.03, 0.23], p = .01) in low walkability and *r* = .17 (95% CI [0.04, 0.31, p < .01) in high walkability was similar, the regression weights differed: In persons living in low walkability, active mobility and social participation were stronger related than in persons living in high walkability (low walkability: *ß* = 0.35, 95% CI [0.14, 0.53], p < .01; high walkability: *ß* = 0.09, 95% CI [-0.01, 0.28], p = .36). The same applied for the prediction of social participation through neighborhood perceptions, with a stronger relation in low walkability vs. high walkability (low walkability: *ß* = 0.27, 95% CI [0.11, 0.42], p < .01; high walkability: *ß* = 0.15, 95% CI [-0.07, 0.32], p = .18). Age was not associated with neighborhood perceptions and active mobility in the low walkability group (neighborhood perceptions: *r* = .10, 95% CI [-0.13, 0.30], p = .50; active mobility: *r* = .05, 95% CI [-0.16, 0.27], p = .62). In contrast, in the high walkability group, age was significantly associated with both variables (*r* = .22 CI [0.06, 0.40], p < .01) and active mobility (*r* = .24 CI [0.04, 0.44] p = .02). The regression of age on social participation was not significant in both groups (low walkability: *ß* = 0.05, 95% CI [-0.12, 0.21], p = .60; high walkability: ß = 0.11, 95% CI [-0.11, 0.32], p = .32) (see Fig. 2).

## Discussion

This cross-sectional study used data from adults living in urban neighborhoods with low and high walkability to investigate and compare overall levels of social participation (operationalized via social activities), active mobility (= walking and biking for transport and recreation), and neighborhood perceptions (= subjectively perceived satisfaction with the neighborhood environment). In addition, the study investigated whether the associations between subjective neighborhood perceptions, active mobility, and social participation differ between individuals who live in low- vs. individuals who live in high walkability neighborhoods. The results show that people living in low and high walkability areas reported equal levels of social participation, active mobility, and neighborhood perceptions. Concerning active mobility, this is surprising, as literature has repeatedly shown higher levels of activity for high than for low walkability areas [59–61]. For example, a study with Belgian adults with a similar age and sex pattern found high walkability to be associated with more active mobility [62]. Similar results stem from a study of 14 cities from 10 different countries from Christiansen, Cerin, Badland, Kerr, Davey, et al. [63], who found cities with more density, parks, street connectivity, and land-use mix, i.e., high walkability areas, to be positively associated with active mobility.

In our study individuals living in low walkability areas were equally satisfied with their neighborhood environment as individuals living in high walkability areas. This is surprising as objectively low walkability areas are characterized by poor connectivity, less land-use mix, fewer amenities, etc. However, the subjective perceptions seem not to be congruent with the objective measure, but to vary between individuals. Our findings are in contrast with those from a study from Australia that investigated individuals’ perceptions concerning different walkability attributes (e.g., land use mix and -access, connectivity, aesthetics, foot/walking paths, etc.) in one neighborhood with high walkability and one with low walkability [64]. The study’s subjects in both neighborhoods were of similar age and had a similar income. Using a modified version of the Neighborhood Environment Walkability Scale (NEWS) that included not identical but similar questions, the study’s findings showed that individuals from low and high walkability perceived their neighborhoods differently: Residents from high walkability constantly reported higher ratings, e.g., regarding the connectivity of streets, land-use mix, or residential density [64]. However, it has to be mentioned that the comparison of studies from Australia and/or North America with studies like this one in a European context has to be considered with caution: Spatial differences, for example in the comparison of city vs. city, cities vs. suburban areas, or in the comparison of cities vs. more rural areas is much greater in Australia, in America, or in the comparison between Australia and America, than it is in Europe. In contrast, the spatial differences are much smaller in the European comparisons. This means that it is important to consider that, if there are no strong differences in the built environmental features in low vs. high walkability neighborhoods, the outcomes of such investigations can’t be expected to reveal differences. Further information about the discrepancies in walkability assessments for Europe vs. Australia/America can be found in a systematic review [38].

The results of equal levels of social participation in low and high walkability are interesting as well because past research identified people to have more social participation if many and varying offerings and characteristics of the environment are present [17, 28, 65]. An explanation for these results could be that instead of the objective walkability influencing individuals’ levels of social participation, their subjective perceptions of the environment play a more important role in influencing their social participation levels. In line with this, the activity levels are equal, with a high average, and might be associated with social participation: The individuals in this study engage a lot in active mobility and through this they might have more possibilities to meet people [22]. For one, while the levels of physical activity (including active mobility) of a third of the population in Europe are lower than the WHO’s recommendation of at least 30 min of moderate physical activity five times per week [66], our sample consisted of active individuals with average active mobility of 35 min per day (note, this average was for 7 days per week). This suggests that active mobility is an integral part of their everyday lives, which seems to be independent of the objective characterization of individuals’ immediate neighborhood environment. Furthermore, the higher levels of active mobility might be associated with individuals’ exposure to environmental features beyond their own neighborhoods, which can also influence health or social outcomes. The subjective perceptions of the individuals living in low walkability are not in line with the objectively determined walkability. And it might be that how individuals perceive their environment is more important in the prediction of their behavior (engaging in active mobility). This is in line with a study by Gebel, Bauman, and Owen [67] that found discordance of a similar direction: Residence living in objectively low walkability who perceived their neighborhood as high walkability reported increased levels of both walking time and positive cognitive attributes compared to residents from objectively high walkability who perceived their neighborhood as low walkability. This shows that when researching the influence of neighborhood environments on individuals’ health behavior, it is important to consider that subjective and objective criteria can vary and lead to different interpretations and that including both measures can prevent false conclusions from being drawn (e.g., [68, 69]). Our findings support the importance of using both objective- and subjective assessment methods when researching the relevance of neighborhood characteristics. But still, more research is needed to understand what influences the subjective perceptions of individuals from different neighborhoods [70, 71].

The high and low walkability groups in our study differed in their age. The persons in the low walkability group were significantly older with a wider variance. However, age was only in the high walkability group a relevant covariate: Being older was related to being more actively mobile and perceiving the neighborhood environment more positively. Age was in both groups not related to social participation. This indicates that with increasing age, the “older” people of this study (mean age: 46,9 ± 16.5 years) more often choose active alternatives to get from A to B and that especially high walkable environments benefit and enable them to do so. Nonetheless, this probably changes again for older people who might have more physical limitations, tend to travel less, and prefer to stay in their immediate surroundings, which they can reach on foot. In line with this thought, older people might appreciate this availability and possibility of being able to engage in active mobility and in turn have a more positive perception of their neighborhood. In line with this, a literature review found multiple associations between positive perceptions of the environment and increased active mobility in older age [72]. Also regarding active mobility, other studies have found high walkability areas to be associated with increased active mobility of older people (e.g., [73, 74]).

The most striking finding is that the prediction of both neighborhood perceptions and active mobility on individuals’ level of social participation was significantly stronger in residential areas with low vs. high walkability. This indicates that residents from low walkability neighborhoods rely more on active mobility to engage in social participation. One explanation is in the context of ‘proximity’, meaning that individuals living in low walkability areas need to travel longer distances to engage in social activities (e.g., visiting bars, clubs, etc.) compared with high walkability areas. This underlines the importance of enabling individuals, especially from low walkability areas, to have the infrastructure and possibility to engage in social participation through active mobility, and in turn, this increases the chance for social participation further [36, 75]. A general way to increase social participation regardless of low or high walkability is for individuals to simply go outside: Research has shown that just being ‘out and about’ in the neighborhood adds to increased active mobility and chances for social participation, and such short, incidental social interactions have additionally been shown to be vital for well-being and health [75, 76]. Similar to active mobility, neighborhood perceptions had a significantly stronger relationship with social participation in low vs. high walkability. This is interesting, as low walkability usually impedes engaging in social participation, and yet, individuals from low walkability were as satisfied with the offerings of their neighborhood as individuals from high walkability. But, it has to be mentioned that social participation was not measured restricted to the neighborhoods the individuals lived in. Therefore, the participants might simply engage in social activities outside their respective neighborhoods. Still, it seems that perceiving the environment as walkable despite the objective lower walkability is associated with higher social participation levels. This underlines the importance of integrating both objective and subjective measures in determining social participation levels. Results from Jun and Hur [69], who found that individuals’ perception of the walkability of their neighborhood environment can stimulate and enhance social interactions, support this. Contrary to the present study (in which social participation was measured via the frequency of engaging in various social activities, regardless of where they take place), Jan and Hur [69] measured the quality of the social interaction in the neighborhood the individuals lived in. Nevertheless, it might be that living in an engaged community with a high sense of cohesion compensates or even extend the effect of a high walkability environment. This could also mean that the objective walkability is less relevant, and that perceived cohesion in a neighborhood - which might be accompanied by a positive perception of the physical neighborhood environment - is of more importance. Another explanation can be that the fewer available offerings fulfill low walkability residents’ needs and that they don’t require additional offerings. This can be seen in line with Mehta [77], who suggested that for people to use streets, the offerings need to be specific to what the individuals who use them need and not random. I.e., the better the fit between residents’ needs and what their environment offers, especially in low walkability, the better their neighborhood perceptions. Another explanation could be that research has indicated a negative relationship between walkability and environmental green, which means that low walkability areas are often more green [78], and more green space has been associated with increased social interactions and individuals feeling less lonely [79, 80]. With regard to that, low walkability areas can not only be seen as “worse” from a subjective point of view but can also be seen as greener, and quieter, because they are not so densely populated with short distances, etc. However, research in this context is scarce, which makes the integration of results difficult and necessitates some broader interpretation.

In sum, individuals’ subjective neighborhood perceptions and levels of active mobility are not always in accordance with and/or can’t be explained via the objectively determined walkability: Despite living in neighborhoods with objectively determined worse environmental conditions, the residents perceive their environment as positive- and have equally high levels of active mobility as individuals who live in neighborhoods with objectively better conditions. Also, the results indicate that those who live in low walkability areas are not a homogeneous group and that living in low walkability areas does not mean that everyone who lives there doesn’t engage a lot in active mobility or social participation. It seems that those who live in low walkability compensate these objectively worse characteristics. In addition, the engagement in social activities was similar in low vs. high walkability areas, which indicates that the objective walkability doesn’t per se affect social participation.

### Strengths and limitations

The integrative analysis of objective walkability, subjective neighborhood perceptions, active mobility, and social participation, is a major strength, as such an approach can reveal important insights into how these variables are associated. An additional strength of this study is the selection of the study areas, which included 12 different neighborhoods (six with low, and six with high walkability) with varying characteristics (e.g., hilly, flat, etc.) to enhance representativeness and generalization and to capture heterogeneity. However, the present paper also has limitations that need to be addressed. First of all, the relatively low number of participants reduced overall power and led to a possibly reduced variability in the study population. Also, despite choosing 12 different low and high walkability neighborhoods, the study participants had an upper-medium SES, making a generalization and transfer of the results to study populations with lower SES difficult. In this context, it is possible that our study population (especially those from the low walkability group) is not representative. In addition, the results of cross-sectional studies don’t allow causal inferences and are a ‘one-time picture’, compared to longitudinal/repeated designs. Also, assessing data via self-report entails the risk of reporting bias and social desirability. Therefore, additional usage of objective measures (e.g., accelerometers to assess active mobility) if applicable is recommended [81, 82].

## Conclusion

The results contribute to the understanding of how active mobility and neighborhood perceptions are related to social participation in low vs. high walkability residential areas. In light of increasing levels of social isolation and loneliness in urban areas, it is important to understand the determinants of social participation and their interdependencies to increase social participation. Especially, as increased levels of social participation have repeatedly been shown to be of additional importance for urban health and livability (e.g., [83, 84]). The result that social participation of individuals living in low walkability areas is stronger related to active mobility and positive neighborhood perceptions has important implications: For example, it can help to demonstrate to city planners and public health officials the importance of enabling active mobility in neighborhoods to promote social participation. However, it would be important to explicitly consider social inequalities when comparing and researching low and high walkability neighborhoods. This also includes a focus on the recruitment of individuals with low SES as they might encounter more barriers to engaging in active mobility and social participation. Also, promoting the possibilities to engage in active mobility and trying to ensure need-specific offerings of amenities, etc. to increase neighborhood perceptions and ultimately social participation can be a feasible and affordable way to promote urban livability and health [75]. Also, besides the benefits of promoting social participation for urban health and livability, the direct positive effects of increased levels of active mobility must not be ignored: Active mobility is beneficial for both physical and mental health, leads to reduced car use, and accompanied improved air quality, and all that contributes concomitantly to more sustainable cities. Another takeaway for city planners and public health officials for their work to create health-enhancing urban areas should be that they need to specifically take into account the subjective perceptions of the residents in addition to objective measures. In sum, accounting for social participation, active mobility, and neighborhood perceptions simultaneously when researching residential areas holds potential to identify ways to create healthy and socially inclusive urban neighborhoods.

### Electronic supplementary material

Below is the link to the electronic supplementary material.

Supplementary Material 1 - Measurement of ‘Active Mobility’

Supplementary Material 2 - Measurement of ‘Neighborhood Perceptions’

Supplementary Material 3 - Measurement of ‘Social Participation’

## Data Availability

The data generated and analyzed during the current study are available via the research data repository of the University of Konstanz, KonDATA: https://dx.doi.org/10.48606/52.

## List of abbreviations

Active mobility: (physically) active mobility (walking cycling for transport and recreation)
Neighborhood perceptions: subjectively perceived satisfaction with the neighborhood environment
