# Supplementary material for: Social participation in the city: exploring the moderating effect of walkability on the associations between active mobility, neighborhood perceptions, and social activities in urban adults
Source: BMC Public Health. 2023 Dec 7;23:2450. doi: 10.1186/s12889-023-17366-0 (PMC10701942; doi:10.1186/s12889-023-17366-0)
Supplement: Supplementary file 1 — Supplementary Material 1 - Measurement of ‘Active Mobility’ [file 12889_2023_17366_MOESM1_ESM.docx]

### Additional file 1

Measurement of ‘Active Mobility’

Question: “On how many days, and for how long have you conducted the following activities in the last four weeks?”

1. ‘Walking to work (also partial sections): On _ days during the 4 weeks and approximately _ minutes per day.’
2. ‘Walking to the grocery store: On _ days during the 4 weeks and approximately _ minutes per day.’
3. ‘Bicycling to work: On _ days during the 4 weeks and approximately _ minutes per day.’
4. ‘Bicycling for other transportation purposes: On _ days during the 4 weeks and approximately _ minutes per day.’
5. ‘Walking for recreation/strolling: On _ days during the 4 weeks and approximately _ minutes per day.’

Note: In the first gap, the participants indicate the number of days on which they have done this activity (ranging from 0 to 31, with 31 indicating that the activity was done every in a month with 31 days). In the second gap, the participants indicate their approximation of the amount of minutes that they did this activity (ranging from 0 to 1440 minutes, with 1440 minutes theoretically representing an activity that was done without a break over the complete course of the day).
