# Supplementary material for: Social participation in the city: exploring the moderating effect of walkability on the associations between active mobility, neighborhood perceptions, and social activities in urban adults
Source: BMC Public Health. 2023 Dec 7;23:2450. doi: 10.1186/s12889-023-17366-0 (PMC10701942; doi:10.1186/s12889-023-17366-0)
Supplement: Supplementary file 2 — Supplementary Material 2 - Measurement of ‘Neighborhood Perceptions’ [file 12889_2023_17366_MOESM2_ESM.docx]

Additional file 3

Measurement of ‘Social Participation’

Question: “How often are you involved in the following activities?”

1. Visit family members/friends.
2. Engage in a hobby outside of home.
3. Attend activities at a community/leisure center.
4. Go shopping.
5. Go to restaurant/pub/café.
6. Attend a sports or cultural event.
7. Take lessons or courses.
8. Participate in a self-help or discussion group.
9. Go to a public library or cultural center.
10. Do some volunteer work.

Note: The participants answered questions 1-10 on a 5-point Likert scale, indicating: 1 (“never”), 2 (“less than once a month”), 3 (“at least once a month”), 4 (“at least once a week”), and 5 (“almost every day”).
