# Supplementary material for: Social participation in the city: exploring the moderating effect of walkability on the associations between active mobility, neighborhood perceptions, and social activities in urban adults
Source: BMC Public Health. 2023 Dec 7;23:2450. doi: 10.1186/s12889-023-17366-0 (PMC10701942; doi:10.1186/s12889-023-17366-0)
Supplement: Supplementary file 3 — Supplementary Material 3 - Measurement of ‘Social Participation’ [file 12889_2023_17366_MOESM3_ESM.docx]

Additional file 2

Measurement of ‘Neighborhood Perceptions’

Questions: “How satisfied are you with…

1. … the accessibility of freeways/highways from your home?
2. … the accessibility of public transport in your neighborhood environment?
3. … the time it takes you to get to work/training/study?
4. … the accessibility of shopping facilities in your neighborhood environment?
5. … the number of friends in your neighborhood environment?
6. … the number of people you know in your neighborhood environment?
7. … the possibility to walk in your neighborhood environment?
8. … the possibility to ride a bike in your neighborhood environment?
9. … the quality of schools in your neighborhood environment?
10. … the accessibility of entertainment facilities in your neighborhood environment (restaurants, cinemas, clubs, etc.)?”

Note: The participants answered questions 1-10 on a 5-point Likert scale, with answers ranging from 1 (“very unsatisfied”) to 5 (“very satisfied”).
